# Purifying and positive selection in the evolution of stop codons

**DOI:** 10.1038/s41598-018-27570-3

**Published:** 2018-06-18

**Authors:** Frida Belinky, Vladimir N. Babenko, Igor B. Rogozin, Eugene V. Koonin

**Affiliations:** 1 National Center for Biotechnology Information, National Library of Medicine, National Institutes of Health, Bethesda, Maryland USA; 2 Institute of Cytology and Genetics, Novosibirsk, Russia

## Abstract

Modes of evolution of stop codons in protein-coding genes, especially the conservation of UAA, have been debated for many years. We reconstructed the evolution of stop codons in 40 groups of closely related prokaryotic and eukaryotic genomes. The results indicate that the UAA codons are maintained by purifying selection in all domains of life. In contrast, positive selection appears to drive switches from UAG to other stop codons in prokaryotes but not in eukaryotes. Changes in stop codons are significantly associated with increased substitution frequency immediately downstream of the stop. These positions are otherwise more strongly conserved in evolution compared to sites farther downstream, suggesting that such substitutions are compensatory. Although GC content has a major impact on stop codon frequencies, its contribution to the decreased frequency of UAA differs between bacteria and archaea, presumably, due to differences in their translation termination mechanisms.

## Introduction

Termination of protein translation occurs when the translating ribosome reaches a stop codon that is recognized by a release factor^1–4^. Each of the three stop codons, UAA, UGA and UAG, is used in all three domains of life, with occasional reassignment of stop codons for amino acid coding, e.g. in the mitochondria of various eukaryotes^5–8^. Bacteria encode 3 release factors, RF1, RF2 and RF3. RF1 recognizes UAA and UAG stop codons, RF2 recognizes UAA and UGA, and RF3 is responsible for the dissociation of RF1 and RF2 after the release of the peptide^4,9^. The ratio between RF1 and RF2 RNA expression and protein abundance has been linked to the ratio between the number of genes terminated with UAG and UGA stop codons (respectively)^10,11^. The frequency of UAA and UGA stop codons strongly depends on the genomic GC-content, whereas the frequency of UAG appears to be independent of the GC content in bacteria^10,12^. These apparent differences in the usage of the stop codons seems to imply that selective factors exist that differentially affect the different stop codons. Indeed, Povolotskaya and colleagues^12^ have hypothesized that UAG is a suboptimal stop codon, with selection acting against it, whereas the frequencies of the other two stop codons can be explained by GC-content^12^. However, others have shown that UAA is more frequent in highly expressed genes, and accordingly, might be considered the optimal stop codon^10,11^. Moreover, distinct trends have been observed for different bacterial taxa, with Proteobacteria and Cyanobacteria showing higher UAA usage in genes with low GC-content and higher UGA usage in genes with high GC-content, whereas Tenericutes and Mollicutes have a high proportion of UAA regardless of the GC-content^10^. Unlike Bacteria, there are only two release factors in Eukaryotes, eRF1, which is homologous to RF1 and RF2 and recognizes all three stop codons, and eRF3, which is required for eRF1 dissociation^13^. In Archaea, only one release factor has been identified, aRF1, a homolog of eRF1, RF1 and RF2, which is assumed to recognize all three stop codons^14^ and has been shown to function when introduced into a eukaryotic translation system^15^.

As a continuation of our previous work on the evolution of start codons^16^, we were interested in systematically assessing the type of selection pressures that affect evolution of stop codons in different life forms, seeking to identify universal and taxon-specific evolutionary factors. In particular, we sought to reveal the connections that might exist between the selection on stops codons and the evolution of the proteins encoded by the respective genes, in an attempt to attain an integrated view of gene evolution. To these ends, we calculated the frequencies of stop codon switches in sets of closely related genomes including 36 bacterial, one archaeal, and 3 eukaryotic ones, with varying GC content, and identified stop codon swaps that appear to be affected by purifying selection, whereas others are neutral, or possibly, could be subject to positive selection. We also show that the known GC-content dependencies of UAA and UGA frequencies hold in all prokaryotes, albeit with significant differences between archaea and bacteria in the midrange of GC-content.

## Methods

### Analysis of stop codon switches in prokaryotes

The sequences of bacterial and archaeal genomes were extracted from the latest release of the ATGC (Alignable Tight Genome Clusters) database^17^. Mutations in protein-coding and non-coding DNA were reconstructed using a parsimony approach to which end triplets of closely related species were analyzed as previously described^16,18^. All the sequences from each ATGC COG (Cluster of Orthologous Genes) were aligned using MAFFT with the –linsi parameter^19^. Only those stop codons that aligned without gaps immediately upstream of the stop were considered in the switch analysis. In 4-fold degenerate sites, the frequency of G to A substitutions (hereinafter, G > A) was used as a control for UGA > UAA and UAG > UAA. Similarly, the A > G frequency was used as a control for UAA > UGA and UAA > UAG. The 4-fold substitution control for UGA > UAG and for UAG > UGA was calculated as $\exp \_{f}_{UGA > UAG}=\exp \_{f}_{UAG > UGA}=2\cdot f{4}_{G > A}\cdot f{4}_{A > G\cdot }$ The standard error for the frequencies was calculated as $SE=\sqrt{pq/n}$.

### Analysis of stop codon switches in eukaryotes

Data sets of protein-coding genes and of aligned 3′ UTRs were obtained for: (1) primates: *Homo sapiens*, *Callithrix jacchus* and *Otolemur garnettii*, (2) Nematodes: *Caenorhabditis briggsae*, *Caenorhabditis remanei*, and *Caenorhabditis elegans*, (3) yeast: *Saccharomyces cerevisiae*, *Saccharomyces paradoxus* and *Saccharomyces mikatae*. Protein-coding sequences for primates and nematodes were downloaded individually for each species from Ensembl and Ensembl Metazoa databases^20,21^, as well as orthology assignments from Ensembl mart^22^. Genes with ‘one to one’ orthology were extracted and aligned using MAFFT with the -linsi algorithm^19^. Non-coding 3′ UTRs were extracted from MAF aligned files obtained from UCSC^23^, based on genome annotations of hg38 and ce11. Yeast coding and non-coding data sets were the same as analyzed previously^18^. Only those stop codons that aligned without gaps immediately upstream of the stop were considered in the switch analysis. The 3′ UTR control and the 4-fold control switches were calculated as indicated above for prokaryotes. The standard error for the frequencies was calculated as $SE=\sqrt{pq/n}$.

### Evolutionary rate and selection strength estimation

For all ATGCs that contained 12 or more genomes and 900 or more genes, the median *dN/dS* for all genome pairs was used as the proxy for the ATGC-specific strength of selection at the protein level^24^. The Codeml program was used to estimate the *dN/dS* values^25^. All genes in each ATGC, were partitioned into 3 sets with different stop codons (UAA, UGA or UAG). For genes with different stop codons, the significance of the differences between the *dN/dS* values was estimated using the Wilcoxon rank sum test.

### Protein abundance data

Integrated protein abundance values for *E. coli* K12 MG1655 were downloaded from PaxDb^26^. Each protein ID was linked to the corresponding RefSeq gene ID, and the stop codon was extracted from the complete *E. coli* K12 MG1655 genome sequence (NC_000913.3). The abundances of proteins encoded by genes ending with UAA, UGA or UAG were compared using Wilcoxon rank sum test.

### Cumulative substitution scores for the sequences around the stop codons

For each ATGC COG, the sequences from positions −9 to +22 (where −1 is the last position of the gene upstream of the stop codon and +1 is the first position of the stop codon) were compared. Genes with less than 20 bases before the next annotated gene were discarded. A score of 0 was assigned to positions containing identical nucleotides in the compared genomes, and a score of 1 was assigned to positions containing different nucleotides. For each ATGC COG, the differences between all pairs were summed per position such that each position received a value of 1 if at least one pair of genomes had a value of 1 in that position. This is a slight underestimation of the actual number of substitutions because no correction was made for possible multiple substitutions in the same position. Genes containing the sequences **UAA**UG, **UGA**UG and A**UGA** (stop codon shown in bold) were excluded to remove potential unannotated gene pairs with overlapping start- and stop codons. Only the COGs with 15 or fewer substitutions in the 31 base window were used for the analysis, to rule out the possibility that non-homologous sequences were compared. Non-homologous sequences might potentially arise due to indels, horizontal gene transfer (HGT), or gene duplication. Randomized 31 base sequences with one constant position (the first position of the stop codon) are equivalent to 30 base sequences with 4 possible bases in each position. These sequences form a binomial distribution with N = 30 and P = 0.25, for which the mean number of matches is NP = 7.5. Accordingly, the mean number of mismatches in a 31 bp window, with one constant position, is 22.25 and the standard deviation is (NP(1−P))^0.5^ = 2.37. Thus,15 matches in a 31 bp window represent a difference from the mean of more than 3 standard deviations, and therefore, most likely, reflect divergence of homologous sequences from the common ancestor. For each specific stop codon or a stop codon switch, the sum of all substitutions in the 31 base windows from all qualifying COGs was calculated. The statistical significance of the differences was estimated using a chi square test, which was performed separately for the coding region (−9 to −1) and the downstream region (+4 to +22) of the stop codon.

### Influence of GC content on stop codon frequencies

Bacterial GC content and stop codon frequencies were calculated from all bacterial genomes in the ATGC database. Archaeal genomes were downloaded from the NCBI genome database^27^. For ~250 archaeal genomes with gene annotations, the GC content and stop codons were extracted and included in the analysis. To quantify the difference between the UAA frequency decrease as a function of GC content in bacteria and archaea, a Kolmogorov-Smirnoff test was performed separately on three GC content windows (GC <0.4, 0.4 < GC <0.6, and GC >0.6).

### Association between start and stop codons

To check for potential association between start and stop codons, and between start and stop codon switches, all ATGC COGs with the same start and stop codons as well as with different start or stop codons were grouped and counted. The significance of association between start and stop codons was inferred using Fisher’s exact test. To check for an association between start and stop codon switches, the expected frequency of start with stop codon switches was calculated as the product of frequency of the start codon switches and that of stop codon switches. The number of occurrences of start with stop codon switches was compared to the expected frequency multiplied by the total number of COGs, using Fisher’s exact test.

### Data availability

All the data used for the present analysis are available in the Supplementary Information or from the authors upon request.

## Results

### Stop codon switches in prokaryotes

**Figure 1:**
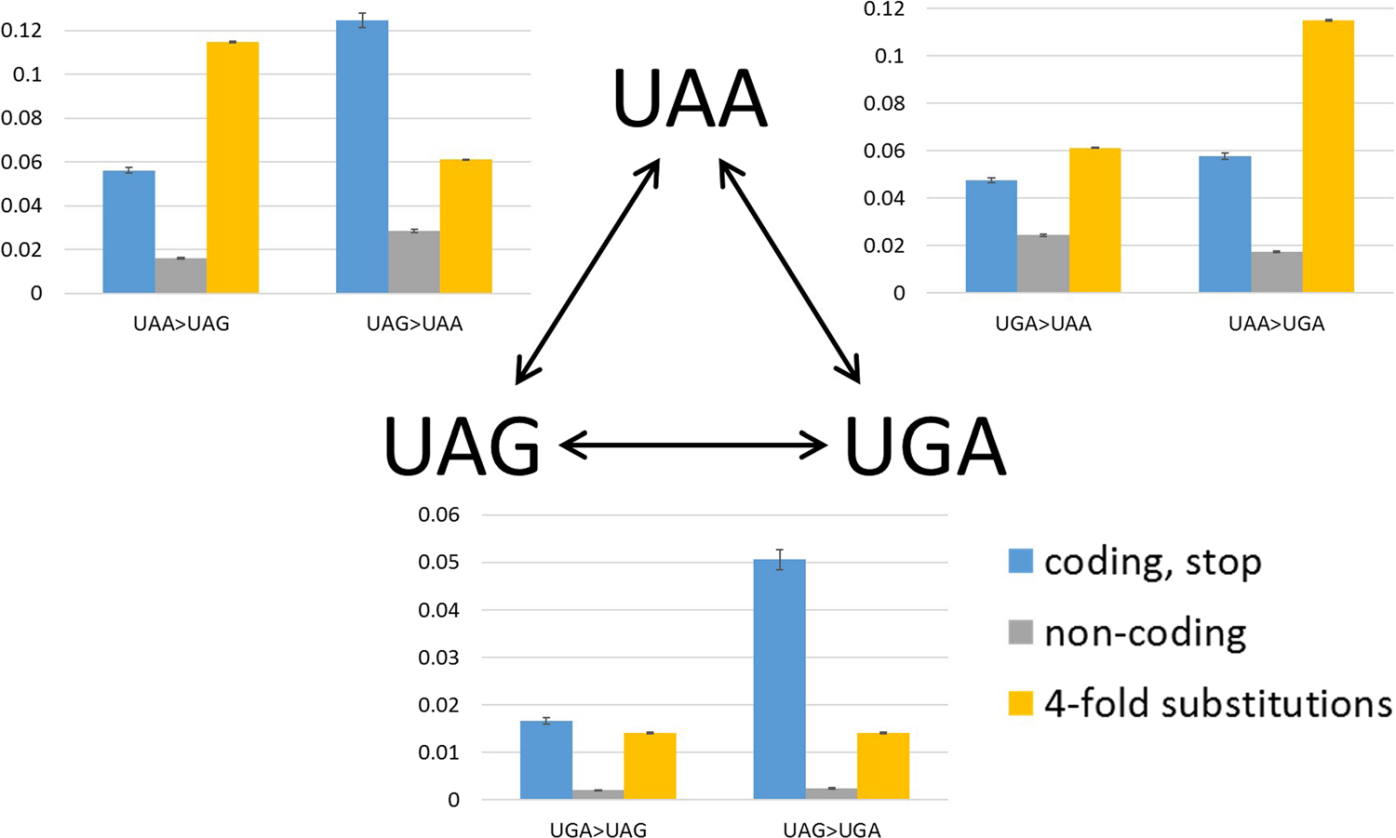
Switches between stop codons in 37 triplets of prokaryotic groups. The switch frequencies in stop codons (blue), the corresponding triplets in intergenic regions (grey) and 4-fold degenerate sites (yellow) are compared.

We examined 37 triplets of closely related genomes from the ATGC database, with confidently determined phylogeny^16,18^, to reconstruct stop codon substitutions (Fig. 1, Table 1). The frequency of stop codon substitutions from a UAG stop codon was significantly greater than the frequency of substitutions to UAG (p = 1.1 × 10^−89^ for UAA, p = 1.6 × 10^−72^ for UGA). Moreover, switches from UAA to UGA were also significantly more frequent than reverse switches from UGA to UAA (p = 9.2 × 10^−9^).

**Table 1:** Stop codon switch counts and frequencies in 37 triples of prokaryotic genomes compared to the switches of the same nucleotide triplets in non-coding regions and 4-fold degenerate sites.

Switch	Ancestral Stop codon count	Stop codon switch Count	Stop codon switch frequency	Ancestral non-coding Scount	Non-coding switch Count	Non-coding switch frequency	Ancestral 4-fold sites count	4-fold sites switch count	4-fold sites switch frequency
UAA > UAG	31,164	1,752	0.0562	135,935	2,201	0.0162	753,203	86,571	0.1149
UAA > UGA		1,798	0.0577		2,358	0.0173			
UGA > UAA	40,595	1,929	0.4752	106,467	2,604	0.0245	2,486,373	152,183	0.0612
UGA > UAG		675	0.0166		210	0.0020			
UAG > UAA	10,375	1,293	0.1246	61,053	1,739	0.0285	261,118^*^	3,674^*^	0.0140^*^
UAG > UGA		525	0.0506		147	0.0024			

*AG and GA counts and frequencies in 4-fold sites are estimated based on the single base frequencies and A > G, G > A switch frequencies. The estimated number of double substitution “AG” to “GA” or vice versa is the product of G > A frequency and A > G frequency multiplied by the estimated number of “GA” sites, times 2.

Examination of these differences at a higher resolution yields a more complex view (Table 2). Thus, UAG is significantly less frequently switched-to, compared to the other two stop codons, only in β-proteobacteria and γ-proteobacteria. In other analyzed groups of prokaryotes, UAG > UGA switches are sometimes significantly more frequent than the reverse UGA > UAG switches (proteobacteria and Actinobacteria) although in other groups (Bacilli, Clostridia, and Methanococci), this is not the case. The general trend seems to be that, in groups where UAA is the most prevalent stop codon (ε-proteobacteria, Bacilli, Clostridia and Methanococci), there are significantly more switches to UAA than from UAA, and no significant difference between switches from UGA to UAG and vice versa. Conversely, in groups where UGA is the dominant stop codon (all other proteobacteria and Actinobacteria), there are significantly more switches to UGA than from UGA, and also significantly more UAG to UAA switches compared to UAA to UAG switches. This pattern implies that UAA is favored by selection in all analyzed groups, even when it is not the dominant stop codon due to the GC content (see below), and that UGA is only favored when GC content is high, but is equivalent to UAG when GC content is low.

**Table 2:** Stop codon switch frequencies in well-sampled prokaryotic phyla.

	UGA>UAG UAG>UGA			UGA>UAA UAA>UGA			UAG>UAA UAA>UAG			Ancestral codon counts		
	#	Freq.	p Fisher	#	Freq.	p Fisher	#	Freq.	p Fisher	UAA	UGA	UAG
α-proteobacteria	161	0.015	5.38e-22	463	0.045	3.82e-17	250	0.142	0.0112	2427	10259	1763
	104	0.059		229	0.094		271	0.112				
β-proteobacteria	145	0.014	5.72e-39	365	0.034	2.36e-21	155	0.112	3.99e-15	2992	10671	1384
	115	0.083		238	0.080		126	0.042				
γ-proteobacteria	193	0.017	5.06e-25	596	0.054	0.0038	286	0.152	9.94e-37	9433	11132	1879
	121	0.064		600	0.064		506	0.054				
δ-proteobacteria	40	0.018	1.29e-06	2	0.001	0.0033	14	0.011	0.0334	54	2273	1254
	60	0.048		2	0.037		3	0.056				
e-proteobacteria	4	0.022	0.1328	38	0.210	0.0247	46	0.365	3.64e-11	550	181	126
	8	0.063		69	0.125		41	0.075				
Bacilii	40	0.016	0.4948	246	0.101	1.88e-31	270	0.135	3.22e-40	9548	2430	2005
	39	0.019		336	0.035		409	0.043				
Clostridia	18	0.025	0.2729	87	0.123	9.91e-06	70	0.177	1.26e-09	1745	709	396
	15	0.038		108	0.062		109	0.062				
Actinobacteria	56	0.024	5.76e-07	41	0.018	7.45e-18	20	0.032	8.21e-07	237	2288	631
	45	0.071		38	0.160		32	0.135				
Methanococci	3	0.034	0.2458	10	0.115	9.26e-04	13	0.144	3.56e-06	1273	87	90
	0	0		37	0.029		29	0.023				

### Frequencies of stop codon switches compared to the frequencies of the same triplet switches in non-coding regions and 4-fold degenerate codon positions

Comparison of the switch frequencies in stop codons with those in the corresponding triplets in non-coding regions (Fig. 1, Table 1) shows that all stop codon switches occur significantly more frequently than the equivalent substitutions in non-coding DNA (p = 5.76 × 10^−197^ for UGA > UAG, p = 1.95 × 10^−293^ for UAG>UGA, p = 2 × 10^−291^ for UAA>UAG, p = 7.97 × 10^−100^ for UGA>UAA, p = 2.89 × 10^−279^ for UAA>UGA, and p = 1.73 × 10^−287^ for UAG>UAA).

To further test whether the frequencies of stop codon switches are indicative of selection, we compared stop codon switch frequencies to the equivalent substitution frequencies in 4-fold degenerate sites (Fig. 1, Table 1). Based on these measurements and calculations, stop codon switches from UAA to a different stop codon are significantly less frequent than anticipated from the comparison to 4-fold degenerate sites (p = 1.48 × 10^−211^ for UAA>UGA and p = 4.87 × 10^−221^ for UAA>UAG). Likewise, UGA>UAA is significantly less frequent than the G>A substitutions in 4-fold degenerate sites (p = 4.41 × 10^−29^). Conversely, the frequency of switches from UAG to another stop codon is significantly higher than in 4-fold degenerate sites (p = 9.59 × 10^−107^ for UAG>UAA and p = 8.36 × 10^−119^ for UAG>UGA). Finally, the UGA>UAG switch is also significantly more frequent than expected from 4-fold degenerate sites albeit to a lesser extent (p = 9.95 × 10^−5^). Assuming neutral evolution of four-fold degenerate positions, we conclude that the slow-changing UAA stop codons are subject to significant purifying selection, in contrast to the fast-changing UAG stop codons that appear to be actively eliminated by positive selection. However, the selection on stop codons appears to be weaker than that in intergenic regions which, in bacteria and archaea, are enriched with regulatory sequences and therefore, to a large extent, subject to purifying selection (Fig. 1; refs^28,29^).

### Stop codons switches in slow-evolving and fast-evolving genes

**Figure 2:**
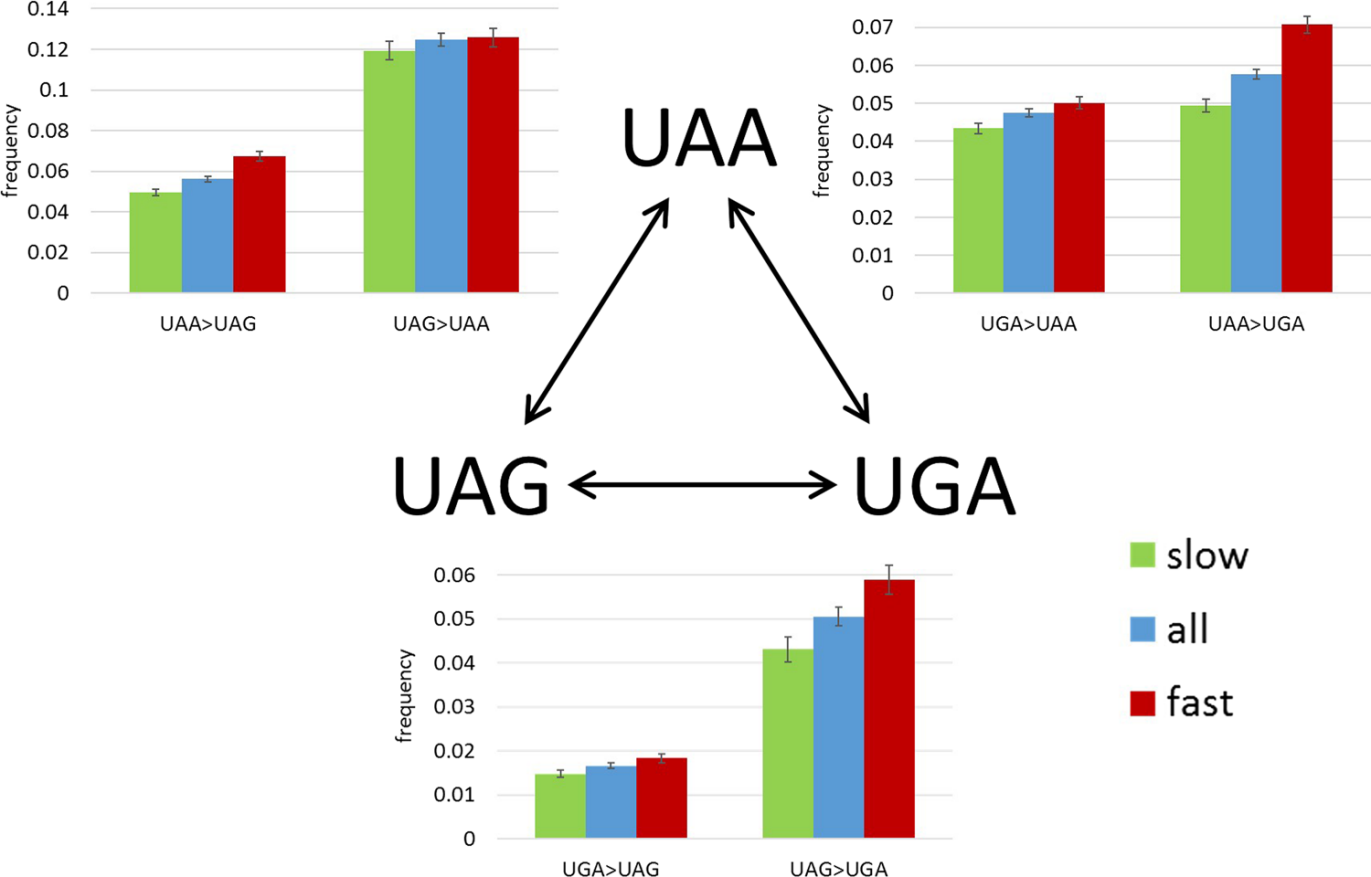
Switches between stop codons in 37 triples of prokaryotic genomes depending on gene evolutionary rate. The switch frequencies are compared for all genes, slow-evolving genes, and fast-evolving genes.

We were further interested in possible links between the stop codon switches and the evolution of the protein encoded by the respective genes, and in particular, whether or not the selective pressure on stop codons tracks that affecting the protein. All genes were divided into slow-evolving and fast-evolving groups, where slow-evolving genes are those with *dN/dS* below the median of the genomic distribution, and fast-evolving genes are those with *dN/dS* above the median for each ATGC triplet (Fig. 2, Table 3). The frequencies of 5 of the 6 possible stop codon switches are significantly higher in fast-evolving genes than in slow-evolving genes (p = 7.66 × 10^−13^ for UAA > UGA, p = 1.56 × 10^−9^ for UAA>UAG, p = 6.74 × 10^−4^ for UAG>UGA, p = 0.0027 for UGA>UAA, and p = 0.0066 for UGA>UAG). The only exception is UAG>UAA for which there is no significant difference between the fast and slow-evolving genes (p = 0.3813). These findings indicate that evolutionary constraints on protein structure and function are mirrored by purifying selection on stop codons.

**Table 3:** Stop codon switch counts and frequencies in 37 triples of prokaryotic genomes separately for slow-evolving vs. fast-evolving genes.

Start codon switches	Slow- evolving genes ancestral stop count	Slow- evolving genes switch count	Slow- evolving genes switch frequency	Fast-evolving genes ancestral stop count	Fast-evolving genes switch count	Fast-evolving genes switch frequency
UAA > UAG	16,665	828	0.0497	12,417	837	0.0674
UAA > UGA		823	0.0494		878	0.0707
UGA > UAA	20,424	887	0.0434	19,310	967	0.0501
UGA > UAG		302	0.0148		354	0.0183
UAG > UAA	4,990	595	0.1192	5,058	636	0.1257
UAG > UGA		215	0.0431		298	0.0589

### Evolutionary rates and protein abundances of genes with different stop codons

The analysis of stop codon switches described above indicates that the choice of the stop codons is not selectively neutral and that the frequency of switches correlates with the evolutionary rates of the respective genes. Accordingly, we further addressed the question whether genes with different stop codons also differed in their evolutionary rates and protein abundance. Gene evolutionary rate and protein abundance are known to be strongly, negatively correlated^28^.

**Figure 3:**
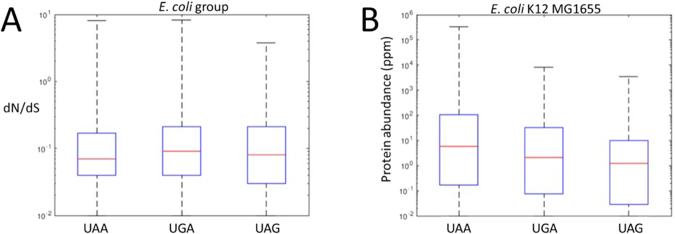
Comparison of the evolutionary rate (**A**) and protein abundance (**B**) in *E. coli* genes ending with UAA, UGA or UAG stop codons.

In ATGC001 (*E. coli* and closely related enterobacteria), the evolutionary rates of genes ending with UAA are, on average, significantly lower than those of genes ending with UGA (Fig. 3A; p = 9.77 × 10^−9^), but the difference compared to genes ending with UAG is borderline (Fig. 3A; p = 0.047). The evolutionary rates of genes ending with UGA were not significantly lower than those of genes ending with UAG (p = 0.099). The same trend was observed in 15 of the other 21 examined ATGCs containing 12 or more genomes and at least 900 genes each (Fig. S1 and Table S1).

Proteins encoded by genes with UAA stop codons are, on average, more abundant than those encoded by genes containing either UGA or UAG (Fig. 3B, p = 5.4 × 10^−9^ and p = 2.5 × 10^−8^, respectively); genes ending with UGA are also more abundant, on average, than genes ending with UAG but to a lesser extent (p = 0.0025).

This analysis further supports the congruence in the evolution of protein-coding sequences and stop codons. Indeed, UAA, the stop codon subject to comparatively strong purifying selection, as shown above, is preferred in slow-evolving genes encoding abundant proteins.

### Coupling of individual stop codons and stop codon switches with the evolution of the surrounding sequences

**Figure 4:**
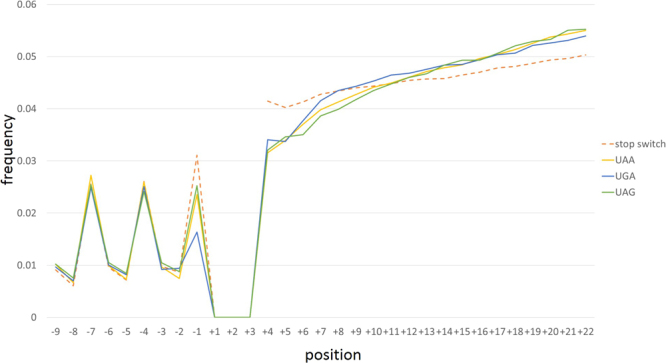
Cumulative substitution frequencies in gene regions adjacent to the stop codons. The cumulative substitution frequencies are shown for genes with UAA stop codons (yellow), UGA stop codon (blue), and UAG stop codon (green) as well as for those with a switch in the stop codon (dashed orange) from >200 prokaryotic ATGC groups.

In genes ending with UAA, UGA or UAG, a consistent trend of cumulative substitution was observed (Fig. 4), where the number of substitutions is gradually increasing with the distance from the stop codon. Comparing the cumulative substitutions in genes ending with different stop codons, all 3 show different frequencies of cumulative substitutions between positions +6 to +11. In this region, genes ending in UGA accumulate significantly more substitutions than genes ending with either UAA of UAG (p = 4.4 × 10^−20^ and 2.1 × 10^−26^, respectively), and genes ending with UAA have slightly more substitutions than genes ending with UAG (p = 0.001). Additionally, position +4 (the base after the stop) accumulates significantly fewer substitutions in genes ending with UAA and UAG compared to genes ending with UGA (p = 9.75 × 10^−09^). Comparison of the genes with no change in the stop codon, with genes with a stop codon switch reveal a highly significant increase (p < 10^−324^) in the frequency of substitutions immediately downstream of the stop codon in genes in the latter group, suggesting compensation between changes in the stop codon and in the immediate downstream region.

### Stop codons switches are strongly impacted by GC content

**Figure 5:**
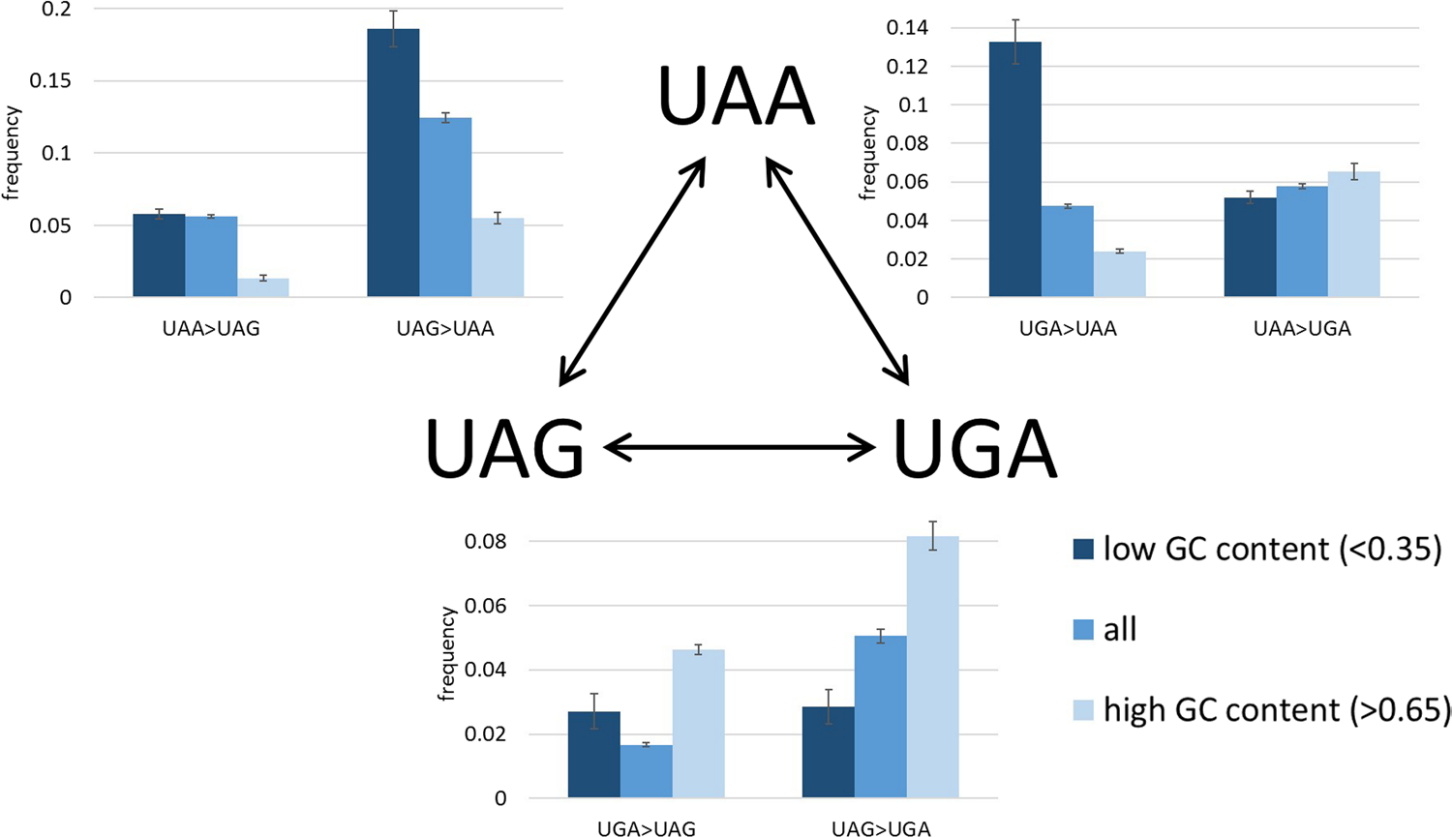
Switches between stop codons in 37 triplets of prokaryotic groups depending on GC content. The switch frequencies are compared between genes with low GC content (GC < = 0.35) and genes with high GC content (GC > = 0.65).

Switches in stop codons toward UAA are significantly more frequent in genomes with low GC content (Fig. 5, p-val = 4.9 × 10^−42^ for UGA>UAA, p-val = 4.1 × 10^−28^ for UAG>UAA), whereas in genomes with a high GC content, there are significantly more switches toward UGA (p-val = 7.2 × 10^−7^ for UAA>UGA, p-val = 8.8 × 10^−6^ for UAG>UGA). Switches from UGA to UAG are significantly less frequent in low GC genomes compared to high GC genomes, albeit to a lesser extent (p-val = 0.002).

**Figure 6:**
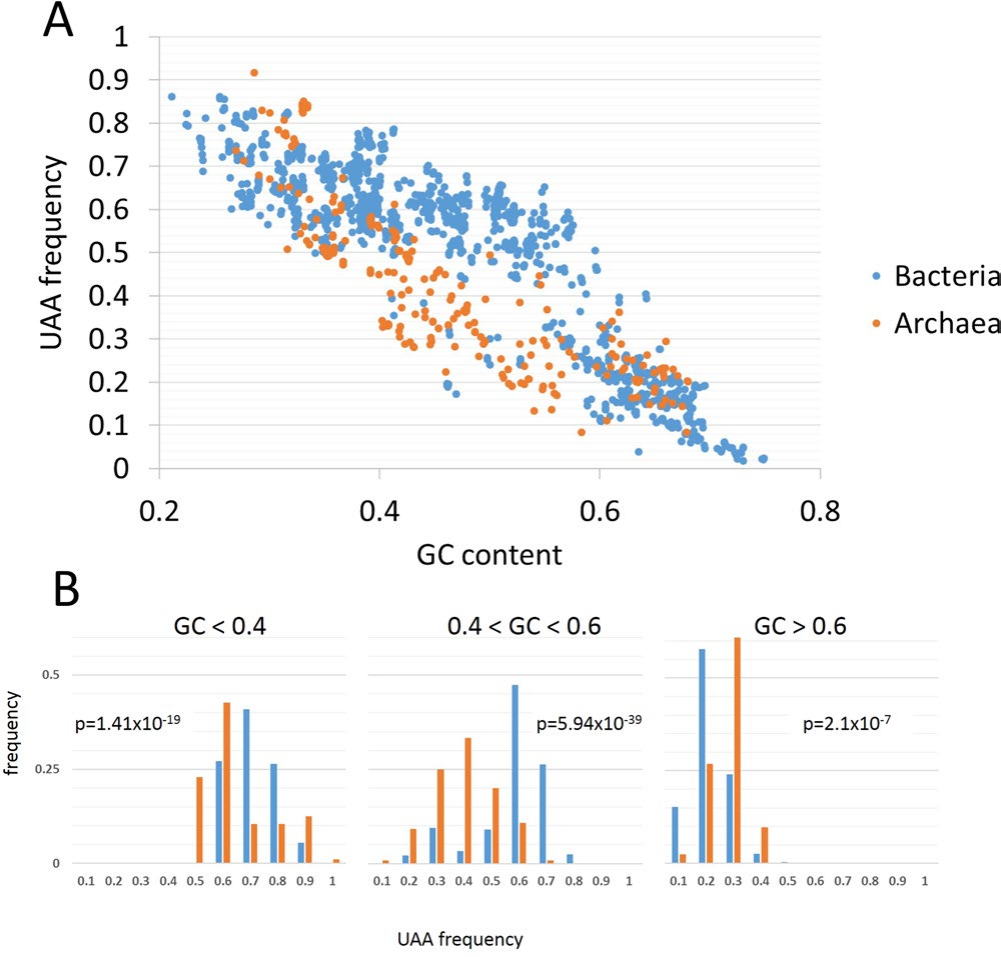
Dependency of the UAA stop codon frequency on GC content. (**A**) UAA frequency vs GC content in bacteria (blue) and archaea (orange). (**B**) Three bins of GC content with distribution of UAA frequencies for each bin in bacteria (blue) and archaea (orange).

Prokaryotic stop codon usage is highly correlated with GC content (Fig. 6). Specifically, in bacteria, UAA usage negatively correlates with GC content (R = −0.876, p < 0.0001) whereas UGA usage correlates positively (R = 0.866, p < 0.0001). A similar trend is observed in archaea, with a negative correlation between UAA usage and GC content (R = −0.843, p < 0.0001) and positive correlation between UGA usage and GC content (R = 0.74, p < 0.0001). However, comparison of the UAA usage in bacteria to that in archaea shows that, although both negatively correlate with GC content, the extent of the decrease in UAA is different between bacteria and archaea (Fig. 6A). The frequencies of genes ending with UAA in bacterial genomes significantly differ from those in archaea (Fig. 6B). The most pronounced difference is the distribution in the mid-range GC content, between 0.4 and 0.6, where bacteria have significantly more genes ending with UAA than archaea (p-val = 5.9 × 10^−39^).

### Association between stop and start codons

Following our recent analysis that demonstrates selection on start codons in prokaryotes^16^, we addressed the possibility of coordinated changes in start and stop codons. Genes starting with AUG or UUG are more likely to end with UAA compared to genes starting with GUG (Table S2; p-val = 3.64 × 10^−96^ and p-val = 1.46 × 10^−96^), which are more likely to end with UGA (Table S2; p-val = 6.66 × 10^−47^ and p-val = 3.08 × 10^−93^). However, genes starting with AUG are also less likely to end with UAA compared to genes starting with UUG (Table S2; p-val = 6.06 × 10^−20^ and p-val = 2.61 × 10^−07^) and are more likely to end with UGA compared to genes starting with UUG (Table S2; p-val = 6.63 × 10^−45^). Finally, genes starting with AUG are less likely to end with UAG (Table S2; p-val = 2.61 × 10^−07^ for UUG and p-val = 5.06 × 10^−28^ for GUG) and there is no significant difference between genes starting with either GUG or UUG for ending with UAG (Table S2; p-val = 0.015). The expected frequency of start and stop codon switches to coincide in the same gene was 0.0554 whereas the observed frequency was 0.0538. Although these frequencies are very close, Fisher’s exact test on the actual counts indicates that significantly fewer cases of double switches were observed than expected (Table S2; p-val = 1.44 × 10^−04^).

### Stop codon switches in eukaryotes

**Figure 7:**
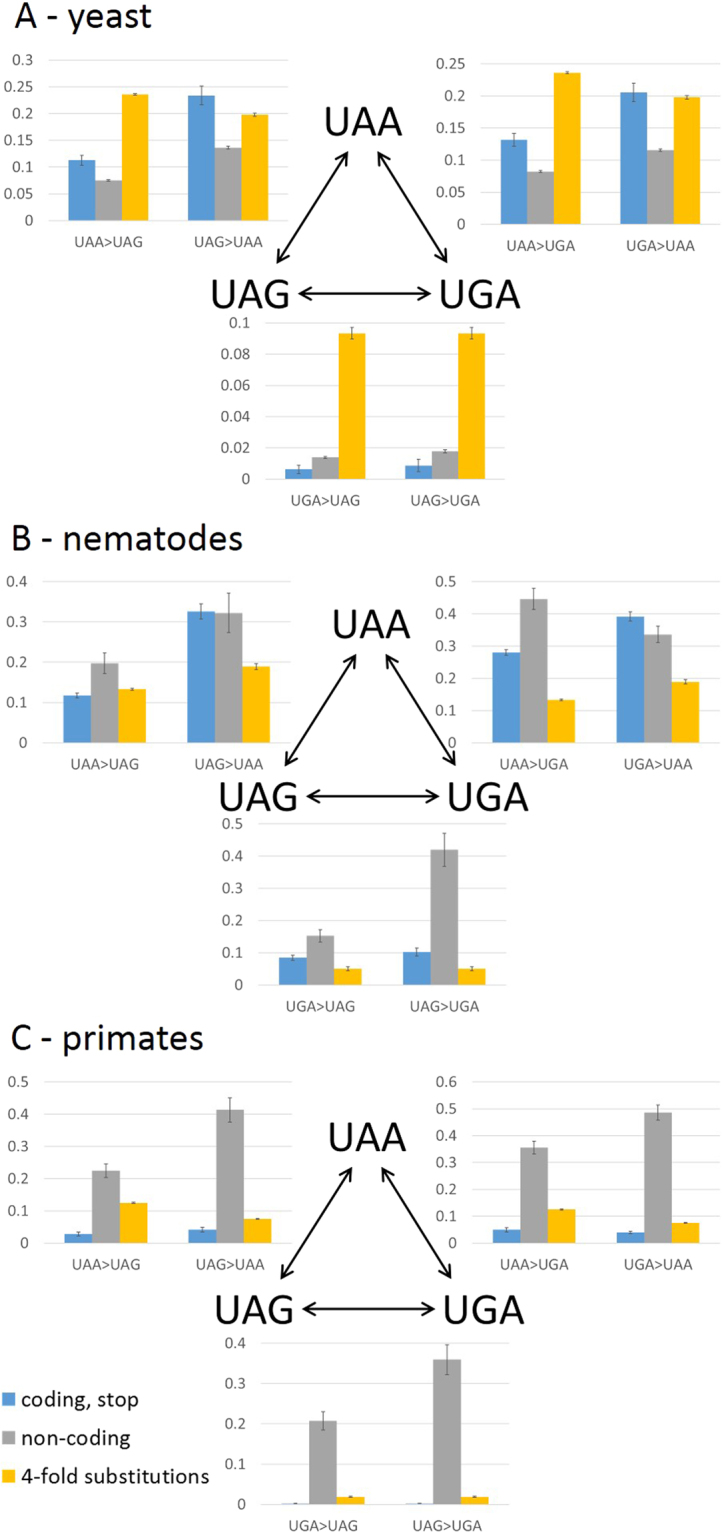
Switches between stop codons in 3 groups of eukaryotes. (**A**) Yeast (**B**) nematodes (**C**) primates. The switch frequencies are compared between stop codons (blue), corresponding triplets in 3′ UTRs (grey) and 4-fold degenerate sites (yellow).

We additionally analyzed stop codon switch frequencies in 3 groups of eukaryotes, namely, yeast, nematodes and primates. Major differences exist between these 3 groups regarding the sequence conservation in non-coding 3′ UTR compared to 4-fold degenerate positions. The 3′ UTRs in yeast are subject to significant purifying selection as shown by comparison of the substitution rate to that in 4-fold degenerate positions and are, in this respect, similar to prokaryotes (Fig. 7A, ^29^). In contrast, in both nematodes and primates, 3′ UTRs evolve significantly faster than 4-fold degenerate sites. Conceivably, in these genomes, the rate of 3′ UTR change is more indicative of neutral evolution, whereas the lower substitution rate in 4-fold degenerate sites is due to various form(s) of purifying selection on synonymous sites. In yeast, the rate of switch frequency from UAA to other stop codons is significantly lower than the substitution rate in 4-fold degenerate sites but significantly higher than the switch rates in 3′ UTRs (Table S3) which is again similar to the trend in prokaryotes and compatible with purifying selection. The yeast switch frequency toward UAA is not significantly different from the rate of 4-fold degenerate substitutions (Table S3). The frequencies of switches that require double substitutions (i.e., UAG< >UGA) is similar to those in 3′ UTRs and significantly less frequent than those in 4-fold degenerate sites (Table S3). In nematodes (Fig. 7B), switches from UAA stop are significantly less frequent than in 3′ UTRs (Table S3). The frequencies of stop codon switches toward UAA are not significantly different from switch frequencies in 3′ UTRs (Table S3). Double switches in stop codons are significantly less frequent than in 3′ UTRs (Table S3). In primates (Fig. 7C), all stop codon switches are less frequent than in 3′ UTRs and than in 4-fold degenerate sites (Table S3), with the largest difference observed between switches from UGA (the major stop codon in primates) compared to 3′UTR.

## Discussion

Several studies have addressed the usage and evolution of the stop codons in bacteria, emphasizing the role of GC content in the choice of the major stop codon and the correlations with the abundance of RF1 and RF2. Here we capture the strong connection between GC content and stop codon usage but, in addition, our analysis scheme allows us to distinguish between mutational and selective factors. The major conclusion from the switch analysis is that UAA is maintained by selection in all analyzed prokaryotes, even in species where the major stop codon is UGA (Tables S2–S4). These results are in agreement with the findings of previous studies suggesting that UAA is the optimal stop codon based on its higher proportion in highly expressed genes^10,11^. Our analysis also provides evidence of positive selection driving the change from UAG to other stop codons, mainly, in ATGCs where the major stop codon is UGA (Tables S2, S5–S6). This observation agrees with the findings of Povolotskaya *et al*.^12^ suggesting that UAG is a suboptimal stop codon. However, in contrast to the conclusions of Povolotskaya and colleagues^12^, we show that UAA is not maintained solely due to the mutational bias towards AU. Rather, the comparison to 4-fold degenerate sites suggests that UAA is maintained by purifying selection. The selection affecting stop codons is relatively weak. In particular, comparison of the strength of purifying selection to that on start codons indicates that purifying selection on UAA is slightly lower than that on GUG and UUG start codons and much weaker than purifying selection on AUG, the primary start codon^16^.

In agreement with previous observations that highly expressed genes most often end with UAA^10,11^, we observed that, across many groups of prokaryotes, genes ending with UAA, on average, encode orthologs of proteins that are highly abundant in *E. coli* and are highly evolutionarily conserved compared to the genes ending with UGA and UAG. Fast-evolving genes generally accrue more stop codon switches than slow-evolving genes, suggesting that the higher evolutionary rate involves also the stop codons. However, the absence of significant difference between the UAG>UAA rates in the fast-evolving and slow-evolving genes indicates that positive selection driving the change from UAG to UAA is even stronger in slow-evolving genes than in fast-evolving ones. The causes of the observed preference for UAA as the stop codon, particularly in highly expressed, slow-evolving genes remain unknown. One potentially plausible possibility is that UAA is less prone to formation of stable secondary structures in RNA molecules than UAG or UGA which facilitates the release factor access and is likely to be particularly relevant for highly expressed genes. Furthermore, the frequency of readthrough differs for the different stop codons and is the highest for UGA, at least, in *E. coli*^30^. Because the deleterious effect of readthrough is the greatest for abundant proteins, the difference in readthrough frequencies could, in part, explain the strong preference for UAA in genes encoding such proteins.

The comparison of the stop codon switch frequencies in different GC content ranges shows that, not unexpectedly, a major impact of low GC content is the increased frequency of switches toward UAA. Similarly, in high GC content genomes, there is a moderate excess of UAA > UGA switches, and UAG > UGA switches. However, as evident from the data for individual ATGCs (Table S4), the excess of UAA > UGA and UGA > UAA in high and low GC content genomes, respectively, results from mutational biases, whereas the difference in the UAG > UGA switches is the result of positive selection.

The impact of GC content is further evident when comparing start and stop codons in the same gene because genes that start with GUG also tend to end with UGA, the preferred stop codon in high GC content. The GUG-UGA association seems most likely to be influenced by GC content but the association between the UUG start and the UAA stop suggests that such genes possess fined tuned signals to reduce protein translation initiation but to increase termination efficiency. Furthermore, the reduced association between AUG and UAG might indicate that genes that are optimized for translation initiation tend to avoid inefficient termination.

The strong impact of GC content on stop codon frequencies has been well documented, and is also linked to the RF1/RF2 abundance ratio^10–12^. Although the decrease in UAA as a function of GC content has been described as a general trend among bacteria^10,12^, we show here a significantly different decrease rate for UAA as a function of the GC content in bacteria vs. archaea. In bacteria, the decline of the UAA content is much less pronounced than it is in archaea for genomes with <60% GC. Conceivably, this difference stems from the differences in the termination process between bacteria and archaea. Whereas bacteria have two release factors, RF1 with the affinity to UAA and UAG, and RF2 with the affinity to UAA and UGA, archaea have only one aRF that recognizes all three stop codons. With the increase in GC content to around 0.5, bacteria show, on average, a stronger preference for UAA (>0.50) than archaea in which the UAA frequency drops to below 0.5. Assuming that the release factor levels need to be adjusted to produce enough RF2 to recognize many genes ending with UGA, maintaining stronger purifying selection on UAA could be a less costly solution to the problem than increasing the abundance of RF2 in the moderate GC content range. However, when the GC content climbs higher than 0.6, purifying selection on UAA would have to be extremely strong to maintain the frequency of this stop codon, so that mutations increasing the RF2 abundance become more likely. Environmental conditions could additionally affect the stop codon bias because some hyperthermophilic bacteria, in particular, those of the genus *Thermotoga*, have GC content of ~0.465 and UAA frequency of 0.17–0.19. These values are closer to those in Archaea than to those in mesophilic bacteria with GC content of 0.4–0.6. However, the other few bacteria with UAA frequencies close to those of archaea with the same GC content are mesophiles which emphasizes the existence of several taxon-specific trends affecting the evolution of stop codons.

The importance of UAA as a stop codon is further supported by the analysis of stop codon switches in eukaryotes. In primates, evidence of purifying selection was obtained for all stop codons although the most frequent one is UGA. In contrast, in nematodes and yeasts, switches from UAA are subject to purifying selection, whereas switches toward UAA appear to be neutral. The double switches in stop codons (UAG< >UGA) in all Eukaryotes appear to be subject to purifying selection as well, in a clear contrast to prokaryotes, where the UGA > UAG switch appears to be mostly neutral, whereas the UAG > UGA switch is subject to positive selection in many groups.

To switch from a UAG to a UGA stop codon or *vice versa*, two nucleotide substitutions are required. The two mutational paths between these stop codons pass either through a UAA stop codon, or through UGG which codes for tryptophan. Changing a stop codon to a coding one inevitably extends the respective protein which is generally expected to be deleterious although the detrimental effect of such mutations can be mitigated by additional stop codons that are often present downstream of the one terminating a given ORF^10^. In the current analysis, no switches from UAG or UGA to UGG were recorded which is most likely due to purifying selection that eliminates such mutations. These considerations seem to provide an explanation for the observations of apparent positive selection affecting the switches from UGA to UAG and back. This effect is likely to stem from the strong positive selection on UGG to UGA and UGG to UAG mutations in cases when UGG is an intermediate in a stop codon switch. Apparently, this selection is strong enough to result in rapid elimination of UGG codons replacing stop codons from microbial populations so that such UGG intermediate become difficult to detect in genomic comparisons. This phenomenon recapitulates the previously proposed scenario for double switches in serine codons where a mutation to an intermediate non-synonymous codon is followed by positive selection which leads to rapid reversal to serine^18^. These findings are also consistent with the previously reported apparent selection against gene overlaps that are likely to emerge as the result of protein extension by mutations changing stop codons to amino acid-coding ones^31–33^.

The gradual increase in the substitution rate with the distance from the stop codon in the downstream region is compatible with the existence of termination regulatory elements in this region. Unlike the Shine-Dalgarno ribosome-binding site that is important for translation initiation in most prokaryotes and is typically located 5-7 bases upstream of the start codon^34^, these putative additional termination signals appear to be most important when they are adjacent to the stop codon, as indicated by the gradual increase in the substitution rate downstream of the stop. This finding is in conflict with the observations on increasing conservation away from the stop codon in bacteria^35^. One downstream element that might affect termination is the U at position +4^31,32^, whereas other studies have suggested that bases farther downstream are involved in termination efficiency^33,36^. Our results support the possibility that downstream termination signals are common and that changes to these signals accompany and compensate stop codon switches in prokaryotes.

## Electronic supplementary material

Supplementary Material
