# Supplementary material for: Purifying and positive selection in the evolution of stop codons
Source: Sci Rep. 2018 Jun 18;8:9260. doi: 10.1038/s41598-018-27570-3 (PMC6006363; doi:10.1038/s41598-018-27570-3)
Supplement: Supplementary file 1 — Supplementary Material [file 41598_2018_27570_MOESM1_ESM.pdf]

Purifying and positive selection in the evolution of stop codons

Frida Belinky[1], Vladimir N. Babenko[2], Igor B. Rogozin[1], Eugene V. Koonin[1*]

[1]National Center for Biotechnology Information, National Library of Medicine, National Institutes of Health, Bethesda, Maryland, USA; [2]Institute of Cytology and Genetics, Novosibirsk, Russia

[*]**To whom correspondence should be addressed. Email:** koonin@ncbi.nlm.nih.gov

Supplementary information

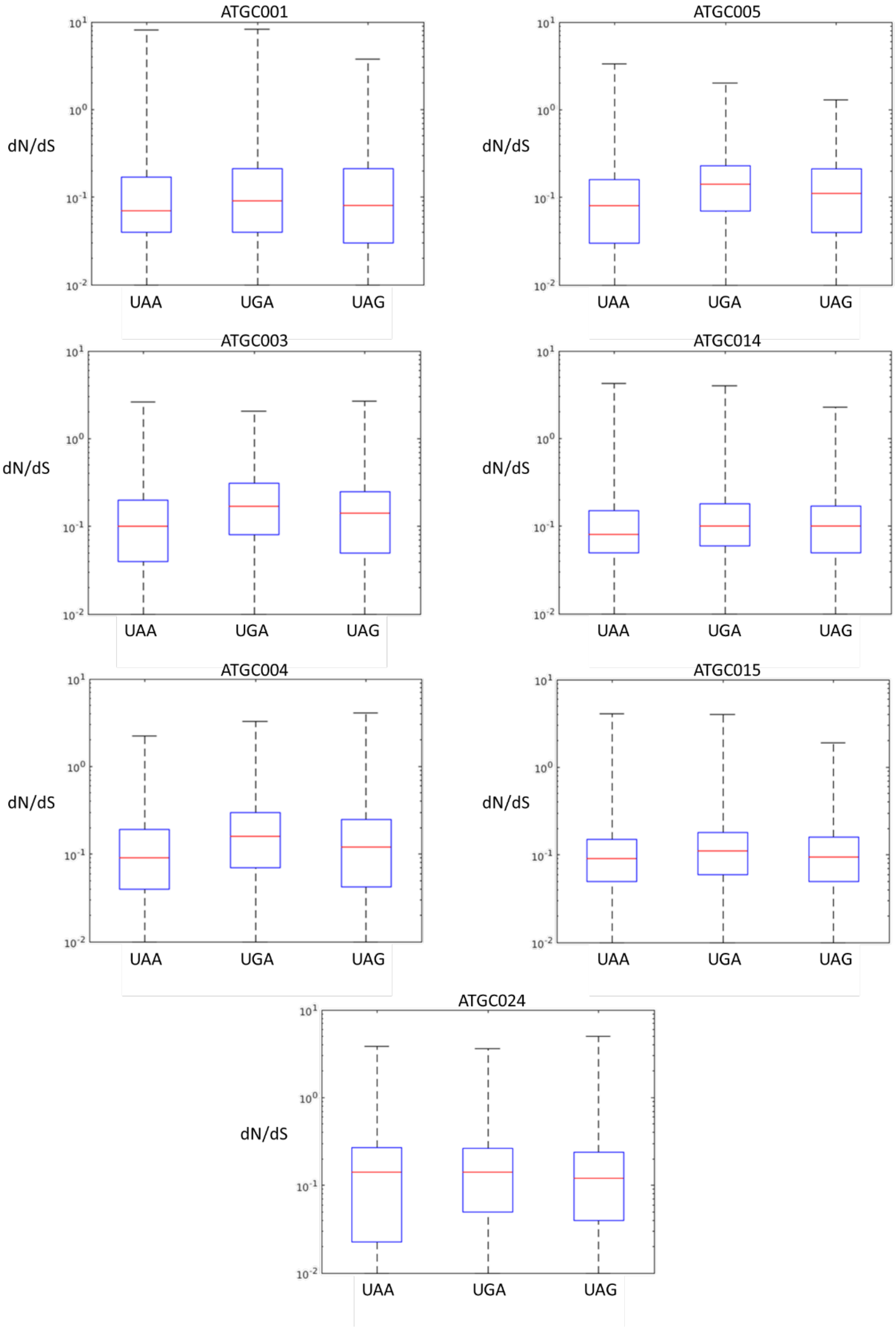

Figure S1. (Part I)

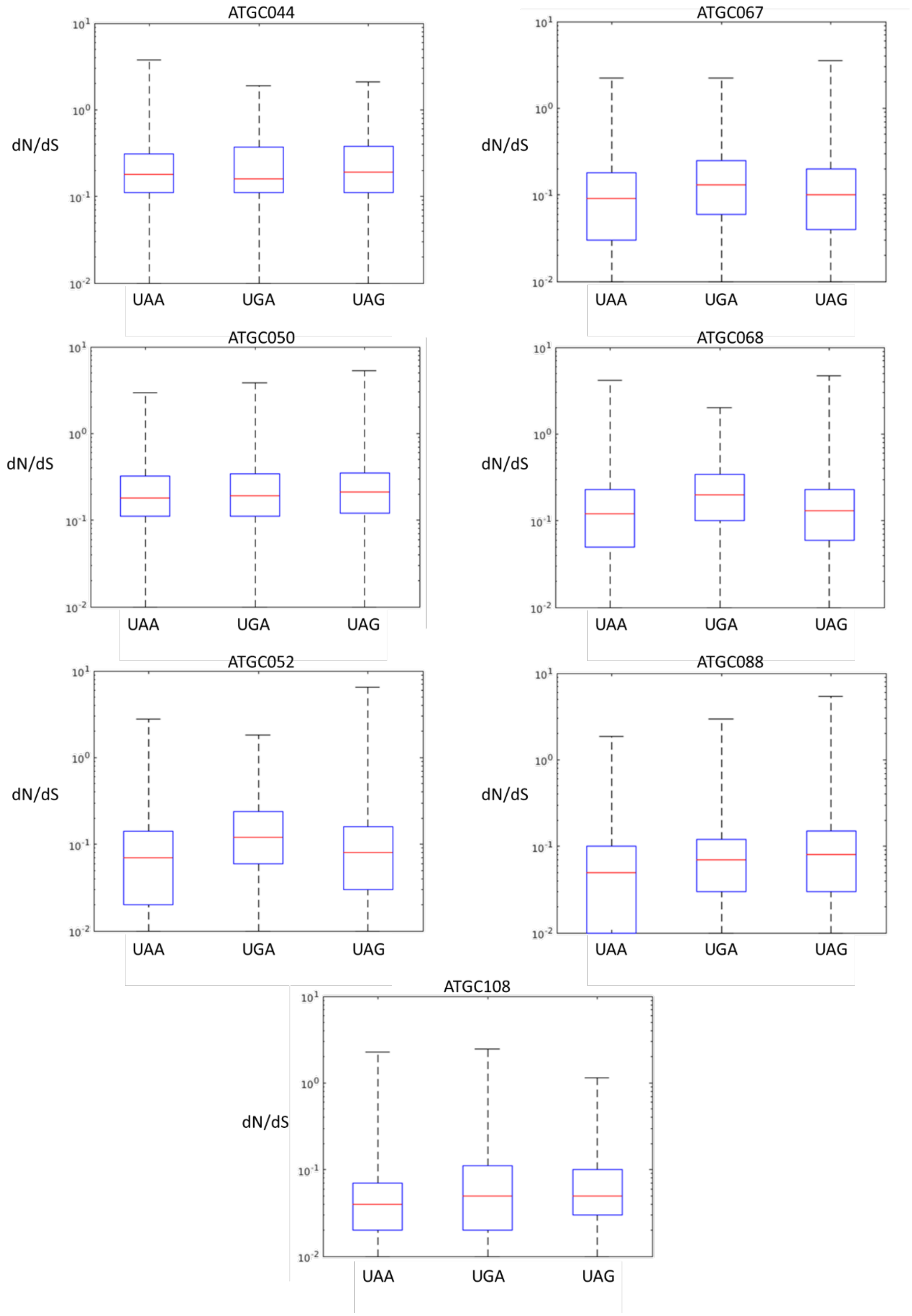

Figure S1. (Part II)

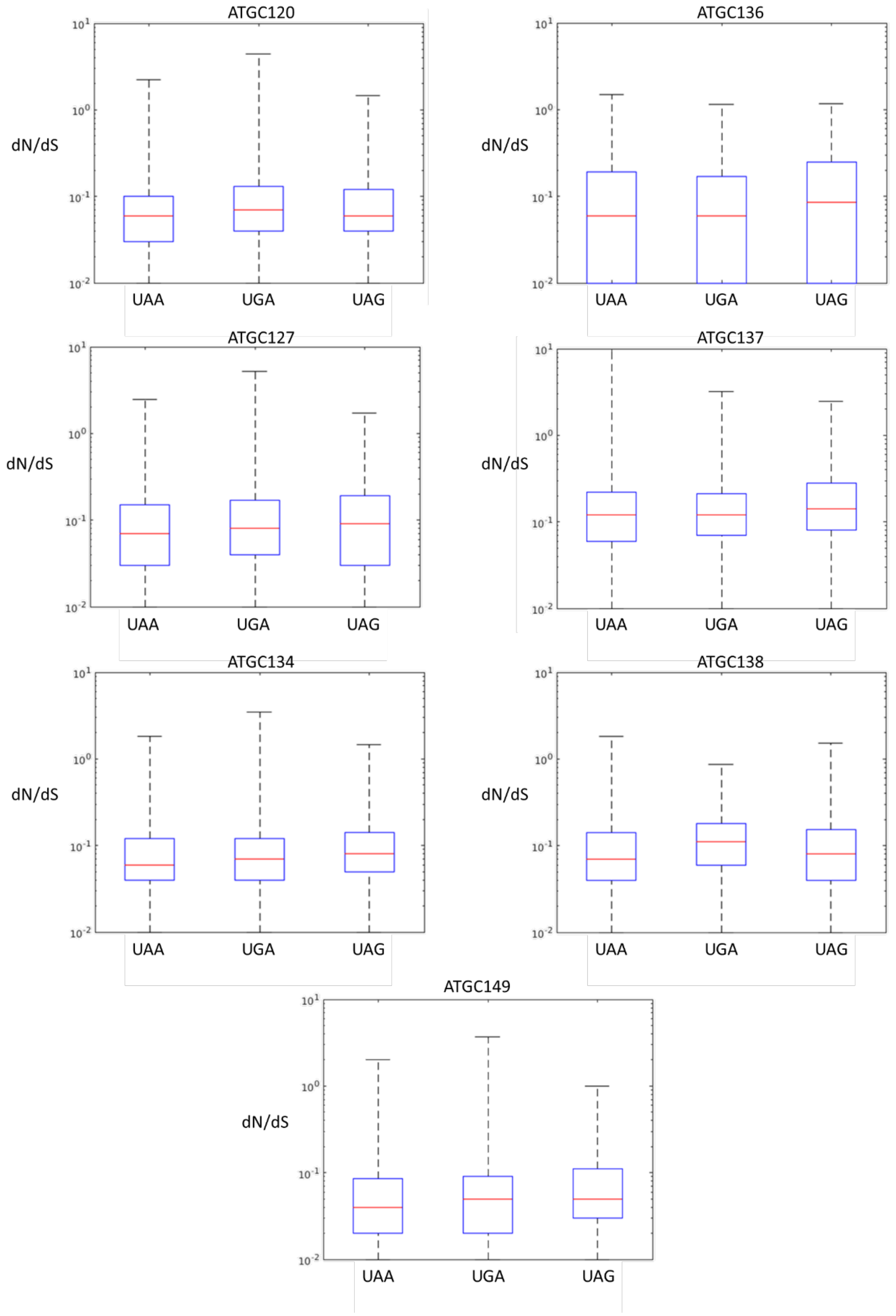

Figure S1. (Part III). Evolutionary rates of genes ending with UAA, UGA and UAG in 21 ATGC groups with more than 12 species and more than 900 genes. The lower bound of *dN/dS* values

is zero. However, to enable presentation in log scale, the lower bound was shifted to a finite value that was arbitrarily set to 0.01.

Table S1. Comparison of the *dN/dS* values for genes ending with UAA, UGA and UAG in 21 ATGC groups. Wilcoxon rank sum test was performed on the dN/dS values of genes ending with different stop codons in each group. Fisher's exact test was performed on the number of genes ending with each codon and having higher or lower dN/dS compared to the overall median in each ATGC group. The ATGC groups included are the ones that have more than 12 species and more than 900 total genes.

ATGC group	# species	median dN/dS of genes ending with			Wilcoxon rank sum test			Fisher's exact test			# genes ending with		
		UAA	UGA	UAG	UAA vs. UGA	UAA vs. UAG	UGA vs. UAG	UAA vs. UGA	UAA vs. UAG	UGA vs. UAG	UAA	UGA	UAG
ATGC001	109	0.07	0.09	0.08	9.77E-09	0.046977	0.099435	2.14E-06	0.007825	0.546799	4288	2617	976
ATGC003	22	0.1	0.17	0.14	8.91E-09	0.000293	0.026085	8.73E-09	2.11E-05	0.083445	958	245	348
ATGC004	22	0.09	0.16	0.12	1.67E-09	0.002399	0.006217	9.74E-08	0.011317	0.004706	941	202	335
ATGC005	16	0.08	0.14	0.11	7.42E-09	0.000598	0.009604	1.20E-06	0.001728	0.024492	753	146	298
ATGC014	31	0.08	0.1	0.1	2.14E-10	5.75E-06	0.09752	1.37E-09	1.92E-06	0.180818	4568	1020	1241
ATGC015	24	0.09	0.11	0.095	1.54E-11	0.027537	0.0041	5.34E-08	0.082796	0.010899	2802	1074	710
ATGC024	32	0.14	0.14	0.12	0.586768	0.375581	0.046846	0.755279	0.096105	0.006722	347	1125	689
ATGC044	40	0.18	0.16	0.19	0.762478	0.236212	0.55047	0.391831	0.149434	0.054203	886	321	373
ATGC050	51	0.18	0.19	0.21	0.365852	0.014537	0.164837	0.364778	0.005692	0.08568	961	463	348
ATGC052	42	0.07	0.12	0.08	5.66E-11	0.021235	8.87E-05	1.08E-08	0.068298	0.000799	1641	226	325
ATGC067	18	0.09	0.13	0.1	9.71E-06	0.062755	0.003002	1.24E-05	0.098387	0.003038	968	244	584
ATGC068	13	0.12	0.2	0.13	7.29E-08	0.514027	2.10E-06	4.88E-07	0.413223	3.88E-05	807	202	475
ATGC088	13	0.05	0.07	0.08	6.12E-07	7.96E-08	0.003921	6.79E-07	2.23E-07	0.04826	771	3328	545
ATGC108	31	0.04	0.05	0.05	0.001657	2.28E-06	0.220232	0.147586	0.00087	0.108203	1708	368	303
ATGC120	14	0.06	0.07	0.06	3.59E-07	0.002611	0.028655	0.000157	0.022185	0.108021	2988	751	1223
ATGC127	19	0.07	0.08	0.09	0.002055	0.000801	0.448065	0.014902	5.55E-05	0.08307	1798	776	492
ATGC134	13	0.06	0.07	0.08	0.002996	7.29E-06	0.002821	0.000296	6.59E-07	0.007558	971	2609	634
ATGC136	19	0.06	0.06	0.085	0.534375	0.429123	0.209882	0.552082	0.830422	0.483296	325	533	134
ATGC137	19	0.12	0.12	0.14	0.366966	0.002036	0.010231	0.514863	0.004755	0.022158	965	715	216
ATGC138	18	0.07	0.11	0.08	0.001525	0.207064	0.038212	0.004547	0.153873	0.067239	881	102	325
ATGC149	18	0.04	0.05	0.05	0.25092	6.84E-05	0.053784	0.405646	0.001382	0.108484	2524	297	342

Table S2. Association between start and stop codons across all ATGC groups, and association between start and stop codon switches

		start with stop	total start	frequency	Fisher's exact test p-val
AUG	UAA	147152	329777	0.446217	6.06E-20
UUG	UAA	9893	19724	0.501572	
AUG	UAA	147152	329777	0.446217	3.64E-96
GUG	UAA	13619	37845	0.359863	
AUG	UGA	127825	329777	0.38761	6.63E-45
UUG	UGA	6219	19724	0.315301	
AUG	UGA	127825	329777	0.38761	6.66E-47
GUG	UGA	16912	37845	0.446875	
AUG	UAG	54800	329777	0.166173	2.61E-07
UUG	UAG	3612	19724	0.183127	
AUG	UAG	54800	329777	0.166173	5.06E-28
GUG	UAG	7314	37845	0.193262	
UUG	UAA	9893	19724	0.501572	1.46E-96
GUG	UAA	13619	37845	0.359863	
GUG	UGA	16912	37845	0.446875	3.08E-93
UUG	UGA	6219	19724	0.315301	
UUG	UAG	3612	19724	0.183127	0.015
GUG	UAG	7314	37845	0.193262	

	start and stop switch	total	frequency	Fisher's exact test p-val
observed	35891	667144	0.053797981	1.44E-04
expected	36945	667144	0.055377849	

Table S3. Significance (p-values) for comparison of stop codon switched to non-coding DNA (intergenic regions in prokaryotes and 3' UTRs in eukaryotes), and to 4-fold degenerate sites substitutions. p-values in red indicated significantly more frequent stop codon switches compared to the control. p-values in blue indicate significantly less frequent stop codon switches compared to the control. Values with grey background indicate the chosen control for a given taxa based on the comparison of non-coding regions to substitutions in 4-fold degenerate sites (the control with more substitutions was chosen as it is assumed to be closer to neutrality).

Start codon switches		primates	nematodes	yeast	prokaryotes
UAA>UAG	Stop vs. 4-fold	$2.39x10^{-16}$	0.0531	$2.08x10^{-18}$	$4.87x10^{-221}$
	Stop vs. non-coding	$3.5x10^{-20}$	0.0036	$3.59x10^{-5}$	$2x10^{-291}$
UAA>UGA	Stop vs. 4-fold	$2.59x10^{-9}$	$1.49x10^{-52}$	$4.59x10^{-13}$	$1.48x10^{-211}$
	Stop vs. non-coding	$8.5x10^{-28}$	$2.86x10^{-4}$	$3.34x10^{-7}$	$2.89x10^{-279}$
UGA>UAA	Stop vs. 4-fold	$3.68x10^{-7}$	$2.06x10^{-24}$	0.66	$4.41x10^{-29}$
	Stop vs. non-coding	$1.5x10^{-58}$	0.2	$5.71x10^{-10}$	$7.97x10^{-100}$
UGA>UAG	Stop vs. 4-fold	$8.67x10^{-9}$	0.0012	$6.15x10^{-23}$	$9.95x10^{-5}$
	Stop vs. non-coding	$1.2x10^{-44}$	0.0015	0.08	$5.76x10^{-197}$
UAG>UAA	Stop vs. 4-fold	0.0015	$7.56x10^{-9}$	0.09	$9.59x10^{-107}$
	Stop vs. non-coding	$4.5x10^{-24}$	1	$2.28x10^{-7}$	$1.73x10^{-287}$
UAG>UGA	Stop vs. 4-fold	$1.31x10^{-4}$	$9.09x10^{-5}$	$1.87x10^{-15}$	$8.36x10^{-119}$
	Stop vs. non-coding	$8.2x10^{-38}$	$5.026x10^{-9}$	0.14	$1.95x10^{-293}$

Table S4. Summary of all selective directions for individual ATGC groups as presented in tables S5-S10. Blue indicated purifying selection, red indicates positive selection. Only significant values after Bonferroni correction are colored. ATGC groups are sorted by frequency of UGA stop codons.

ATGC	Major stop codon		UAA>UGA	UAA>UAG	UAG>UAA	UAG>UGA	UGA>UAA	UGA>UAG
	stop codon	frequency						
149	UAA	0.842224	1.34E-53	8.01E-25	0.117123	0.153318	1.16E-05	0.066027
108	UAA	0.749449	5.96E-38	4.74E-41	6.91E-06	0.049209	3.02E-11	1.98E-06
14	UAA	0.73769	1.47E-24	3.91E-12	0.02306	0.015015	9.93E-08	0.020152
15	UAA	0.655584	1.57E-09	3.21E-15	0.492716	0.084499	0.354924	0.508529
1	UAA	0.653701	2.43E-27	2.35E-50	0.000303	1	0.077937	0.02242
100	UAA	0.605396	8.88E-83	5.39E-62	3.53E-07	4.06E-05	0.055865	3.8E-08
199	UAA	0.578614	3.37E-27	3.73E-16	0.959798	8.77E-07	0.002216	1.16E-05
97	UAA	0.55814	1.85E-11	1.38E-22	7.62E-11	0.004177	0.182561	0.015191
111	UGA	0.597144	1.13E-14	3.75E-19	0.025713	0.011478	0.778997	0.025942
252	UGA	0.634739	0.766339	1	0.020417	4.12E-18	0.000534	2.16E-07
125	UGA	0.675275	0.061497	0.563893	2.87E-28	1.78E-15	0.368206	5.83E-06
188	UGA	0.687789	7.52E-11	1.6E-19	1.14E-07	5.76E-09	0.428442	0.56655
123	UGA	0.691943	9.84E-06	0.022403	1.75E-06	0.000217	0.862002	0.781535
71	UGA	0.71683	3.24E-12	5.4E-35	0.34601	5.67E-09	0.03009	0.036854
134	UGA	0.733756	0.011943	9.29E-08	0.002236	7.85E-06	0.574732	0.693261
89	UGA	0.739064	3.64E-18	2.02E-36	3.33E-07	5.05E-10	0.157288	0.124547
189	UGA	0.740195	0.000455	3.3E-07	2.54E-08	8.4E-14	0.685579	0.007737
88	UGA	0.760252	3.43E-14	3.4E-24	8.79E-14	5.52E-10	0.003091	0.504506
234	UGA	0.764498	0.043213	0.249847	0.568694	1.38E-12	1.65E-05	1.35E-06
135	UGA	0.765684	2.07E-18	1.29E-25	7.67E-05	6.74E-12	9.56E-05	0.137622
213	UGA	0.83312	0.175246	0.000818	0.076029	0.004225	0.145381	0.211751
165	UGA	0.912687	0.079282	0.00054	0.038496	0.000399	0.026385	1

Table S5. Comparison of UAA>UAG stop codon switches to non-coding and 4 fold degenerate sites in individual ATGCs with at least 2000 genes. Significant p-values, marked in red, are after Bonferroni correction for α=0.05, n=132.

			coding			non-coding			4 fold degenerate sites			p-Fisher
ATGC	Ancestral	switched to	Switch count	Ancestral count	switch frequency	Switch count	Ancestral count	switch frequency	Switch count	Ancestral count	switch frequency	coding vs. 4 fold
149	UAA	UAG	120	2242	0.0535	255	13282	0.0192	5573	43923	0.1269	8.01E-25
108	UAA	UAG	39	1699	0.0230	11	558	0.0197	6614	55186	0.1198	4.74E-41
14	UAA	UAG	113	2472	0.0457	113	11198	0.0101	5425	63848	0.0850	3.91E-12
15	UAA	UAG	61	2031	0.0300	49	7612	0.0064	3651	49295	0.0741	3.21E-15
1	UAA	UAG	65	1731	0.0376	76	7211	0.0105	3199	18682	0.1712	2.35E-50
100	UAA	UAG	104	1683	0.0618	180	9959	0.0181	6357	25577	0.2485	5.39E-62
199	UAA	UAG	151	1737	0.0869	213	8277	0.0257	6124	36893	0.1660	3.73E-16
97	UAA	UAG	86	1176	0.0731	98	5055	0.0194	2517	12906	0.1950	1.38E-22
111	UAA	UAG	26	884	0.0294	18	5204	0.0035	536	4113	0.1303	3.75E-19
252	UAA	UAG	3	54	0.0556	2	530	0.0038	85	1416	0.0600	1
125	UAA	UAG	88	493	0.1785	51	2276	0.0224	2064	10721	0.1925	0.5638927
188	UAA	UAG	27	438	0.0616	29	1185	0.0245	732	2627	0.2786	1.60E-19
123	UAA	UAG	69	654	0.1055	49	3463	0.0141	1428	10072	0.1418	0.0224029
71	UAA	UAG	29	786	0.0369	42	3227	0.0130	3193	14667	0.2177	5.40E-35
134	UAA	UAG	44	457	0.0963	14	866	0.0162	1224	5754	0.2127	9.29E-08
89	UAA	UAG	24	788	0.0305	20	2178	0.0092	945	4323	0.2186	2.02E-36
189	UAA	UAG	28	534	0.0524	29	2338	0.0124	348	2595	0.1341	3.30E-07
88	UAA	UAG	27	595	0.0454	30	1979	0.0152	770	3310	0.2326	3.40E-24
234	UAA	UAG	1	34	0.0294	4	490	0.0082	215	1920	0.1120	0.2498471
135	UAA	UAG	32	474	0.0675	22	1655	0.0133	2080	6467	0.3216	1.29E-25
213	UAA	UAG	7	187	0.0374	3	1464	0.0020	161	1338	0.1203	0.0008175
165	UAA	UAG		114	0.0000	1	448	0.0022	92	1203	0.0765	0.0005398

Table S6. Comparison of UAA>UGA stop codon switches to non-coding and 4 fold degenerate sites in individual ATGCs with at least 2000 genes. Significant p-values, marked in red, are after Bonferroni correction for α=0.05, n=132.

			coding			non-coding			4 fold degenerate sites			p-Fisher
ATGC	Ancestral	switched to	Switch count	Ancestral count	switch frequency	Switch count	Ancestral count	switch frequency	Switch count	Ancestral count	switch frequency	coding vs. 4 fold
149	UAA	UGA	58	2242	0.0259	274	13282	0.0206	5573	43923	0.1269	1.34E-53
108	UAA	UGA	44	1699	0.0259	9	558	0.0161	6614	55186	0.1198	5.96E-38
14	UAA	UGA	75	2472	0.0303	157	11198	0.0140	5425	63848	0.0850	1.47E-24
15	UAA	UGA	79	2031	0.0389	72	7612	0.0095	3651	49295	0.0741	1.57E-09
1	UAA	UGA	116	1731	0.0670	82	7211	0.0114	3199	18682	0.1712	2.43E-27
100	UAA	UGA	69	1683	0.0410	180	9959	0.0181	6357	25577	0.2485	8.88E-83
199	UAA	UGA	114	1737	0.0656	188	8277	0.0227	6124	36893	0.1660	3.37E-27
97	UAA	UGA	125	1176	0.1063	161	5055	0.0318	2517	12906	0.1950	1.85E-11
111	UAA	UGA	35	884	0.0396	24	5204	0.0046	536	4113	0.1303	1.13E-14
252	UAA	UGA	2	54	0.0370	3	530	0.0057	85	1416	0.0600	0.76634
125	UAA	UGA	75	493	0.1521	40	2276	0.0176	2064	10721	0.1925	0.0615
188	UAA	UGA	48	438	0.1096	21	1185	0.0177	732	2627	0.2786	7.52E-11
123	UAA	UGA	50	654	0.0765	34	3463	0.0098	1428	10072	0.1418	9.84E-06
71	UAA	UGA	80	786	0.1018	35	3227	0.0108	3193	14667	0.2177	3.24E-12
134	UAA	UGA	70	457	0.1532	21	866	0.0242	1224	5754	0.2127	0.01194
89	UAA	UGA	59	788	0.0749	18	2178	0.0083	945	4323	0.2186	3.64E-18
189	UAA	UGA	40	534	0.0749	20	2338	0.0086	348	2595	0.1341	0.00045
88	UAA	UGA	49	595	0.0824	42	1979	0.0212	770	3310	0.2326	3.43E-14
234	UAA	UGA	0	34	0.0000	6	490	0.0122	215	1920	0.1120	0.04321
135	UAA	UGA	47	474	0.0992	23	1655	0.0139	2080	6467	0.3216	2.07E-18
213	UAA	UGA	15	187	0.0802	3	1464	0.0020	161	1338	0.1203	0.17525
165	UAA	UGA	3	114	0.0263	0	448	0.0000	92	1203	0.0765	0.07928

Table S7. Comparison of UAG>UAA stop codon switches to non-coding and 4 fold degenerate sites in individual ATGCs with at least 2000 genes. Significant p-values, marked in red, are after Bonferroni correction for α=0.05, n=132.

			coding			non-coding			4 fold degenerate sites			p-Fisher
ATGC	Ancestral	switched to	Switch count	Ancestral count	switch frequency	Switch count	Ancestral count	switch frequency	Switch count	Ancestral count	switch frequency	coding vs. 4 fold
149	UAG	UAA	32	183	0.1749	166	4227	0.0393	4242	17780	0.2386	0.11712
108	UAG	UAA	15	212	0.0708	14	543	0.0258	4905	24185	0.2028	6.91E-06
14	UAG	UAA	67	444	0.1509	137	4826	0.0284	4062	20018	0.2029	0.02306
15	UAG	UAA	33	396	0.0833	40	3332	0.0120	5658	59153	0.0957	0.49272
1	UAG	UAA	36	202	0.1782	78	2536	0.0308	7986	90537	0.0882	0.0003
100	UAG	UAA	81	311	0.2605	129	3870	0.0333	13476	102010	0.1321	3.53E-07
199	UAG	UAA	120	553	0.2170	168	4006	0.0419	12829	58592	0.2190	0.9598
97	UAG	UAA	51	196	0.2602	74	1740	0.0425	6498	78632	0.0826	7.62E-11
111	UAG	UAA	13	188	0.0691	11	2785	0.0039	2398	70595	0.0340	0.02571
252	UAG	UAA	14	1254	0.0112	4	1013	0.0039	1072	191023	0.0056	0.02042
125	UAG	UAA	127	689	0.1843	50	1713	0.0292	6341	117866	0.0538	2.87E-28
188	UAG	UAA	34	306	0.1111	18	1090	0.0165	3743	100670	0.0372	1.14E-07
123	UAG	UAA	55	451	0.1220	47	1960	0.0240	7356	127615	0.0576	1.75E-06
71	UAG	UAA	23	311	0.0740	40	2723	0.0147	11476	188549	0.0609	0.34601
134	UAG	UAA	21	215	0.0977	18	1016	0.0177	4225	91776	0.0460	0.00224
89	UAG	UAA	25	232	0.1078	21	1549	0.0136	6273	203351	0.0308	3.33E-07
189	UAG	UAA	36	400	0.0900	24	2191	0.0110	3865	131118	0.0295	2.54E-08
88	UAG	UAA	44	358	0.1229	26	1379	0.0189	4758	160373	0.0297	8.79E-14
234	UAG	UAA	5	498	0.0100	4	1069	0.0037	918	63329	0.0145	0.56869
135	UAG	UAA	29	273	0.1062	26	2161	0.0120	7206	161741	0.0446	7.67E-05
213	UAG	UAA	16	335	0.0478	2	1075	0.0019	2831	95267	0.0297	0.07603
165	UAG	UAA	5	120	0.0417	4	516	0.0078	1303	87138	0.0150	0.0385

Table S8. Comparison of UAG>UGA stop codon switches to non-coding and 4 fold degenerate sites in individual ATGCs with at least 2000 genes. Significant p-values, marked in red, are after Bonferroni correction for α=0.05, n=132.

			coding			non-coding			4 fold degenerate sites			p-Fisher
ATGC	Ancestral	switched to	Switch count	Ancestral count	switch frequency	Switch count	Ancestral count	switch frequency	Switch count*	Ancestral count**	switch frequency	coding vs. 4 fold
149	UAG	UGA	6	183	0.0328	14	4227	0.0033	591	9769	0.0605	0.153318
108	UAG	UGA	4	212	0.0189	2	543	0.0037	802	16501	0.0486	0.049209
14	UAG	UGA	6	444	0.0135	9	4826	0.0019	572	16592	0.0345	0.015015
15	UAG	UGA	10	396	0.0253	2	3332	0.0006	391	27608	0.0142	0.084499
1	UAG	UGA	6	202	0.0297	6	2536	0.0024	461	15271	0.0302	1
100	UAG	UGA	4	311	0.0129	16	3870	0.0041	1330	20254	0.0657	4.06E-05
199	UAG	UGA	12	553	0.0217	13	4006	0.0032	1671	22987	0.0727	8.77E-07
97	UAG	UGA	15	196	0.0765	7	1740	0.0040	357	11079	0.0322	0.004177
111	UAG	UGA	6	188	0.0319	0	2785	0.0000	28	3162	0.0089	0.011478
252	UAG	UGA	60	1254	0.0478	0	1013	0.0000	1	1379	0.0007	4.12E-18
125	UAG	UGA	58	689	0.0842	3	1713	0.0018	166	8018	0.0207	1.78E-15
188	UAG	UGA	29	306	0.0948	3	1090	0.0028	52	2518	0.0207	5.76E-09
123	UAG	UGA	20	451	0.0443	1	1960	0.0005	117	7147	0.0163	0.000217
71	UAG	UGA	31	311	0.0997	8	2723	0.0029	320	12058	0.0265	5.67E-09
134	UAG	UGA	17	215	0.0791	2	1016	0.0020	102	5185	0.0196	7.85E-06
89	UAG	UGA	21	232	0.0905	1	1549	0.0006	61	4552	0.0135	5.05E-10
189	UAG	UGA	30	400	0.0750	2	2191	0.0009	20	2509	0.0079	8.40E-14
88	UAG	UGA	27	358	0.0754	3	1379	0.0022	47	3428	0.0138	5.52E-10
234	UAG	UGA	29	498	0.0582	2	1069	0.0019	5	1410	0.0032	1.38E-12
135	UAG	UGA	36	273	0.1319	6	2161	0.0028	173	6042	0.0287	6.74E-12
213	UAG	UGA	10	335	0.0299	0	1075	0.0000	8	1062	0.0072	0.004225
165	UAG	UGA	5	120	0.0417	0	516	0.0000	3	1126	0.0023	0.000399

* Switch count for 4 fold degenerate sites was estimates by the product of G>A and A>G frequencies and the total number of sites.

** Ancestral count for 4 fold degenerate sites was estimated by the product of G and A frequencies and total number of sites.

Table S9. Comparison of UGA>UAA stop codon switches to non-coding and 4 fold degenerate sites in individual ATGCs with at least 2000 genes. Significant p-values, marked in red, are after Bonferroni correction for α=0.05, n=132.

			coding			non-coding			4 fold degenerate sites			p-Fisher
ATGC	Ancestral	switched to	Switch count	Ancestral count	switch frequency	Switch count	Ancestral count	switch frequency	Switch count	Ancestral count	switch frequency	coding vs. 4 fold
149	UGA	UAA	24	237	0.1013	217	6299	0.0344	4242	17780	0.2386	1.16E-05
108	UGA	UAA	20	356	0.0562	14	874	0.0160	4905	24185	0.2028	3.02E-11
14	UGA	UAA	39	435	0.0897	154	5261	0.0293	4062	20018	0.2029	9.93E-08
15	UGA	UAA	56	671	0.0835	136	6195	0.0220	5658	59153	0.0957	0.354924
1	UGA	UAA	78	715	0.1091	140	4949	0.0283	7986	90537	0.0882	0.077937
100	UGA	UAA	83	786	0.1056	230	7408	0.0310	13476	102010	0.1321	0.055865
199	UGA	UAA	115	712	0.1615	313	6572	0.0476	12829	58592	0.2190	0.002216
97	UGA	UAA	72	735	0.0980	168	3972	0.0423	6498	78632	0.0826	0.182561
111	UGA	UAA	51	1589	0.0321	36	6283	0.0057	2398	70595	0.0340	0.778997
252	UGA	UAA	2	2273	0.0009	1	1770	0.0006	1072	191023	0.0056	0.000534
125	UGA	UAA	143	2458	0.0582	49	4227	0.0116	6341	117866	0.0538	0.368206
188	UGA	UAA	54	1639	0.0329	40	2525	0.0158	3743	100670	0.0372	0.428442
123	UGA	UAA	140	2482	0.0564	84	5290	0.0159	7356	127615	0.0576	0.862002
71	UGA	UAA	140	2777	0.0504	39	4861	0.0080	11476	188549	0.0609	0.03009
134	UGA	UAA	79	1852	0.0427	31	2320	0.0134	4225	91776	0.0460	0.574732
89	UGA	UAA	75	2889	0.0260	30	3701	0.0081	6273	203351	0.0308	0.157288
189	UGA	UAA	82	2661	0.0308	38	3802	0.0100	3865	131118	0.0295	0.685579
88	UGA	UAA	120	3022	0.0397	37	3222	0.0115	4758	160373	0.0297	0.003091
234	UGA	UAA	6	1727	0.0035	6	1489	0.0040	918	63329	0.0145	1.65E-05
135	UGA	UAA	69	2441	0.0283	29	4303	0.0067	7206	161741	0.0446	9.56E-05
213	UGA	UAA	91	2606	0.0349	22	3159	0.0070	2831	95267	0.0297	0.145381
165	UGA	UAA	23	2446	0.0094	1	1300	0.0008	1303	87138	0.0150	0.026385

Table S10. Comparison of UGA>UAG stop codon switches to non-coding and 4 fold degenerate sites in individual ATGCs with at least 2000 genes. Significant p-values, marked in red, are after Bonferroni correction for α=0.05, n=132.

ATGC	Ancestral	switched to	coding			non-coding			4 fold degenerate sites			p-Fisher
			Switch count	Ancestral count	switch frequency	Switch count	Ancestral count	switch frequency	Switch count*	Ancestral count**	switch frequency	coding vs. 4 fold
149	UGA	UAG	7	237	0.0295	16	6299	0.0025	591	9769	0.0605	0.066027
108	UGA	UAG	1	356	0.0028	2	874	0.0023	802	16501	0.0486	1.98E-06
14	UGA	UAG	6	435	0.0138	10	5261	0.0019	572	16592	0.0345	0.020152
15	UGA	UAG	7	671	0.0104	3	6195	0.0005	391	27608	0.0142	0.508529
1	UGA	UAG	11	715	0.0154	6	4949	0.0012	461	15271	0.0302	0.02242
100	UGA	UAG	16	786	0.0204	27	7408	0.0036	1330	20254	0.0657	3.80E-08
199	UGA	UAG	22	712	0.0309	21	6572	0.0032	1671	22987	0.0727	1.16E-05
97	UGA	UAG	12	735	0.0163	10	3972	0.0025	357	11079	0.0322	0.015191
111	UGA	UAG	5	1589	0.0031	4	6283	0.0006	28	3162	0.0089	0.025942
252	UGA	UAG	40	2273	0.0176		1770	0.0000	1	1379	0.0007	2.16E-07
125	UGA	UAG	94	2458	0.0382	5	4227	0.0012	166	8018	0.0207	5.83E-06
188	UGA	UAG	29	1639	0.0177	5	2525	0.0020	52	2518	0.0207	0.56655
123	UGA	UAG	38	2482	0.0153	13	5290	0.0025	117	7147	0.0163	0.781535
71	UGA	UAG	54	2777	0.0194	8	4861	0.0016	320	12058	0.0265	0.036854
134	UGA	UAG	33	1852	0.0178	3	2320	0.0013	102	5185	0.0196	0.693261
89	UGA	UAG	27	2889	0.0093	2	3701	0.0005	61	4552	0.0135	0.124547
189	UGA	UAG	44	2661	0.0165		3802	0.0000	20	2509	0.0079	0.007737
88	UGA	UAG	35	3022	0.0116	6	3222	0.0019	47	3428	0.0138	0.504506
234	UGA	UAG	41	1727	0.0237		1489	0.0000	5	1410	0.0032	1.35E-06
135	UGA	UAG	55	2441	0.0225	9	4303	0.0021	173	6042	0.0287	0.137622
213	UGA	UAG	11	2606	0.0042		3159	0.0000	8	1062	0.0072	0.211751
165	UGA	UAG	7	2446	0.0029		1300	0.0000	3	1126	0.0023	1

* Switch count for 4-fold degenerate sites was estimates by the product of G>A and A>G frequencies and the total number of sites.

** Ancestral count for 4-fold degenerate sites was estimated by the product of G and A frequencies and total number of sites.
